# A Stress-Induced Bias in the Reading of the Genetic Code in *Escherichia coli*

**DOI:** 10.1128/mBio.01855-16

**Published:** 2016-11-15

**Authors:** Adi Oron-Gottesman, Martina Sauert, Isabella Moll, Hanna Engelberg-Kulka

**Affiliations:** a Department of Microbiology and Molecular Genetics, IMRIC, The Hebrew University-Hadassah Medical School, Jerusalem, Israel; b Department of Microbiology, Max F. Perutz Laboratories, Center for Molecular Biology, Immunobiology and Genetics, University of Vienna, Vienna, Austria

## Abstract

*Escherichia coli mazEF* is an extensively studied stress-induced toxin-antitoxin (TA) system. The toxin MazF is an endoribonuclease that cleaves RNAs at ACA sites. Thereby, under stress, the induced MazF generates a stress-induced translation machinery (STM), composed of MazF-processed mRNAs and selective ribosomes that specifically translate the processed mRNAs. Here, we further characterized the STM system, finding that MazF cleaves only ACA sites located in the open reading frames of processed mRNAs, while out-of-frame ACAs are resistant. This in-frame ACA cleavage of MazF seems to depend on MazF binding to an extracellular-death-factor (EDF)-like element in ribosomal protein bS1 (bacterial S1), apparently causing MazF to be part of STM ribosomes. Furthermore, due to the in-frame MazF cleavage of ACAs under stress, a bias occurs in the reading of the genetic code causing the amino acid threonine to be encoded only by its synonym codon ACC, ACU, or ACG, instead of by ACA.

## IMPORTANCE

The genetic code is a universal characteristic of all living organisms. It defines the set of rules by which nucleotide triplets specify which amino acid will be incorporated into a protein. Our results represent the first existing report on a stress-induced bias in the reading of the genetic code. We found that in *E. coli*, under stress, the amino acid threonine is encoded only by its synonym codon ACC, ACU, or ACG, instead of by ACA. This is because under stress, MazF generates a stress-induced translation machinery (STM) in which MazF cleaves in-frame ACA sites of the processed mRNAs.

## INTRODUCTION

Toxin-antitoxin (TA) systems consist of a pair of genes that encode two components: a toxin and an antitoxin that interferes with the activity of the toxin. In recent years, a lot of attention has been focused on the fact that there is such an abundance of these systems in the chromosomes of most bacteria (1–4). Among these systems in bacterial chromosomes, the first one discovered (5) and the one most studied is the *Escherichia coli* TA system *mazEF* (6–8). It encodes two proteins, the labile antitoxin, MazE, which is degraded by the protease ClpPA, and the stable toxin, MazF (5). Both *mazE* and *mazF* are coexpressed and negatively autoregulated at the transcriptional level (9).

*E. coli mazEF* is triggered by various stressful conditions, including treatment with antibiotics, affecting transcription or translation (10); severe amino acid starvation, leading to an increase in the concentration of ppGpp (5); treatment with mitomycin C or nalidixic acid (NA), leading to DNA damage (11, 12); and high temperatures (10).

Initially, MazF was reported as being a sequence-specific endoribonuclease that preferentially cleaves single-stranded mRNAs at ACA sequences and thereby inhibits protein synthesis (6, 7). Surprisingly, we have subsequently shown that this inhibition is not complete: though MazF inhibits the synthesis of most proteins (about 90%), it selectively enables the specific synthesis of about 10% of proteins (13). The underlying molecular mechanism leading upon MazF induction to the selective translation of a particular set of mRNAs in *E. coli* has been elucidated (14). This molecular mechanism is based on a new form of translation machinery, generated by MazF induction under stressful conditions, termed the stress-induced translation machinery (STM) (14). (i) MazF generates mRNAs by cleaving at ACA sites immediately adjacent (upstream) to (14), or further upstream from, the AUG start codons of specific mRNAs (15), and (ii) MazF targets an ACA site in the 16S rRNA within the 30S ribosomal subunit at the decoding center, thereby removing 43 nucleotides from the 3′ terminus including the anti-Shine-Dalgarno (anti-SD) sequence region (14). These stress ribosomes are selectively able to translate the generated processed mRNAs. Thus, under stressful conditions, when MazF is induced, a novel “MazF regulon” is generated that is translated by the novel “stress ribosomes,” thereby producing “stress proteins.”

Here, we further studied the *E. coli* STM system by constructing a green fluorescent protein (GFP) reporter molecule consisting of a leaderless GFP mRNA that is expressed upon the induction of MazF. Surprisingly, GFP was expressed in spite of the existence of 17 ACA sites in the GFP leaderless mRNA. However, we noticed that all of them are located out of the open reading frame (ORF) of GFP. In contrast, inserting an ACA site in the open reading frame of GFP prevented its expression after *mazF* induction. We also showed, by direct experiments, that MazF cleaves ACA sites only when they are located in the open reading frame (called here frame 0) of the leaderless mRNAs; they were never cleaved when they were located out of frame (designated here frame +1 and frame +2). In addition, the in-frame MazF cleavage of leaderless GFP mRNA was dependent on MazF binding to NNW, an extracellular-death-factor (EDF)-like sequence in the ribosomal protein bS1 (bacterial S1). EDF is the *E. coli* extracellular quorum-sensing pentapeptide NNWNN (16), which mediates bacterial cell death by inducing MazF (17).

## RESULTS

### GFP reporter systems of the STM and their expression dependency on MazF induction.

**FIG 1:**
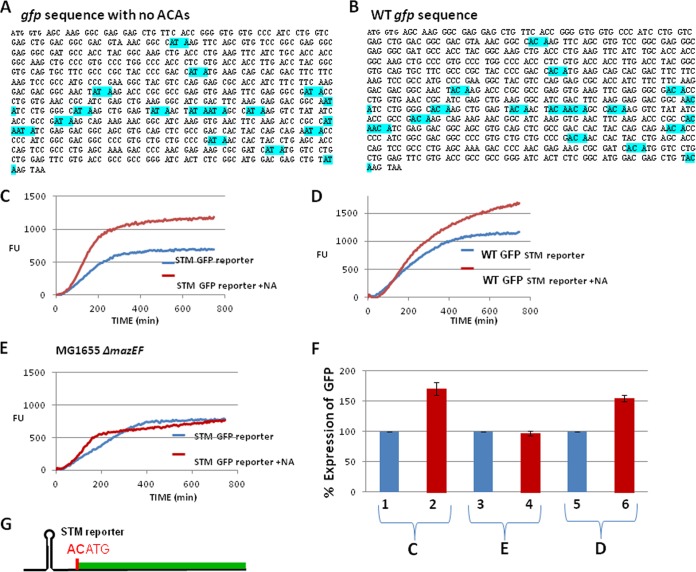
Construction of GFP-STM reporters and the dependency of their GFP expression on MazF induction. (A) *gfp* sequence in which ACA sites were here changed by us without changing the original amino acid (highlighted in blue). (B) Sequence of WT *gfp* (ACA sites are highlighted in blue). (C) GFP expression in *E. coli* strain MG1655 transformed with plasmid pUH-C carrying a GFP-STM reporter with no ACAs in the *gfp* sequence. FU, fluorescence units. (D) As in panel C, but the WT GFP-STM reporter includes ACAs. (E) As in panel C, but in derivative strain MG1655Δ*mazEF*. These results shown are the averages of results from three repeated experiments for each condition. The assays were carried out as described in Materials and Methods. (F) Quantitative comparison of GFP expression in MazF-induced samples (red bars) versus uninduced samples (blue bars). These data were calculated as percentages based on the results shown in panels C, D, and E. For each assay, 100% represents the results for the untreated sample. (G) Schematic presentation of the GFP-STM reporter molecule that we constructed. Immediately after the first ATG, the GFP sequence is either the WT *gfp* that includes ACAs (B) or the sequence that we modified so that it no longer includes any ACAs (A).

In order to investigate the STM system, we used GFP reporters located on the pUH-C plasmid. First, we studied the MazF effect on the expression of the canonical GFP containing the Shine-Dalgarno (SD) region upstream of the AUG start codon. Here, we observed a complete inhibition of GFP expression when nalidixic acid (NA) was applied in order to induce MazF in *E. coli* MG16554 cells (see Fig. S1 in the supplemental material). This inhibition was expected due to the mRNA interferase activity of MazF (6, 7), cleaving ACA sites located in GFP mRNA. To study the STM, we constructed an STM-specific GFP reporter molecule: the first ATG of the *gfp* sequence was preceded by AC, generating an ACATG sequence that would potentially enable MazF to cleave at ACA, thus generating a leaderless GFP mRNA. In addition, this ACATG sequence is preceded by a stem-and-loop structure that interfered with the SD recognition sequence (Fig. 1G). Furthermore, since *gfp* has 17 ACA sites (Fig. 1B), we modified all the ACA sites without changing the original amino acid (Fig. 1A). We inserted this reporter molecule into plasmid pUH-C, which we used to transform *E. coli* MG1655 (wild type [WT]) or its derivative MG1655Δ*mazEF*. At logarithmic phase, we induced *mazF* by adding NA. Adding NA led to a significant increase in GFP expression in the WT strain MG1655 (Fig. 1C and F) but not in MG1655Δ*mazEF* (Fig. 1E and F), confirming that our constructed GFP molecule was indeed a reporter for MazF-induced STM. The induced MazF produced a leaderless GFP mRNA and also generated deficient ribosomes that lacked the last 43 nucleotides of the 16S rRNA, including the anti-SD sequence (14). Note that the increase of the GFP level in the NA-treated WT strain occurred (Fig. 1C) although cell growth was arrested under such conditions (see Fig. S2).

We also studied MazF-dependent *gfp* expression using an STM reporter carrying the WT *gfp* sequence harboring 17 out-of-frame ACA sites (Fig. 1B). We expected that the presence of these ACA sites would cause the *gfp* leaderless mRNA to be cleaved by MazF, induced by the addition of NA, leading to less expression of *gfp* than in the untreated, control culture. We were surprised to observe that in this case the level of GFP was higher than that in the untreated control culture (Fig. 1D). Thus, here MazF generated a leaderless GFP mRNA and a deficient ribosome but did not act as a GFP mRNA interferase. Furthermore, similar levels of GFP were obtained by *mazF* induction of the two STM reporter systems, one containing the WT *gfp* sequence with ACAs (Fig. 1D and F) and the other containing the *gfp* sequence with no ACAs (Fig. 1C and F). Using the strains MG1655 and MG1655Δ*mazEF*, we found that the expression of the WT *gfp* reporter was dependent on *mazF* induction because there was no increase in GFP levels in strain MG1655Δ*mazEF* (see Fig. S3 in the supplemental material).

### ACA sites located in frame 0 of the *gfp* sequence interfere with MazF-induced STM-GFP expression.

**FIG 2:**
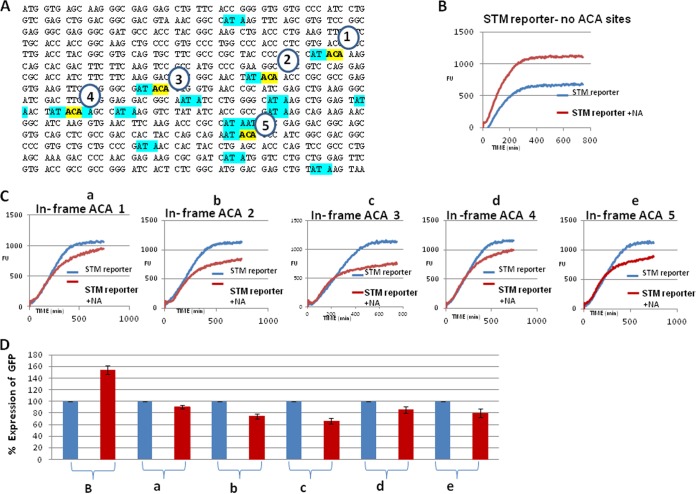
Introducing ACAs in reading frame 0 of the STM-GFP reporter led to reduced MazF-induced GFP expression. (A) Locations of five different in-frame ACA sites in the *gfp* sequence of the STM reporter. We used the STM-GFP reporter with no ACA sites as a platform (Fig. 1A). Each ACA was inserted at a different location, generating only one ACA site in frame 0. The modified triplets are highlighted in yellow and indicated by circles with the numbers 1 to 5. The various sites of the triplets modified to be ACA were selected to be in place of or adjacent to the original out-of frame ACA sites (highlighted in blue in the WT *gfp* sequence [Fig. 1B]) that we had originally modified (Fig. 1A). (B) Expression of the GFP-STM reporter with no ACAs in the *gfp* sequence (as in Fig. 1B). (C) Expression of the GFP-STM reporter with one of the five ACA sites at five different locations in frame 0 of the *gfp* sequence (a to e correspond to locations 1 to 5 as described for panel A). The assays were carried out as described in the legend to Fig. 1. (D) Quantitative comparison (percent) of the results in panels B and C was carried out as described in the legend to Fig. 1F.

Finding that, in spite of the presence of 17 ACA sites, the WT GFP reporter was resistant to MazF cleavage led us to inspect these sites more carefully: each of the 17 ACA sites was located in the +1 frame and not one was in frame 0. In response, we asked whether MazF would act similarly if the ACA sites were located in frame 0 of the *gfp* sequence in the STM reporter. To this end, into the *gfp* sequence of our STM reporter (which harbored no ACAs [Fig. 1A]), we inserted an ACA in each of five different frame 0 sites. We selected five locations (Fig. 2A, circled 1 to 5) in which we modified the sequence so that an ACA now in frame 0 would be adjacent to one of the frame +1 ACAs of the WT *gfp* sequence. We found that for each of these artificially introduced frame 0 STM reporters, the level of GFP expression was reduced in the treated WT strain (Fig. 2C, a to e) while it was unaffected in the Δ*mazEF* derivative (see Fig. S4 in the supplemental material). In contrast, when we used an STM-GFP reporter with a *gfp* sequence that had no ACAs, the addition of NA led to an increase in the level of MazF-mediated GFP expression (Fig. 2A). By quantitative comparison, we clearly show that rather than an increase of about 50% of the MazF-mediated expression of the STM reporter with no ACAs (Fig. 2D, first two bars on the left marked B), there was a decrease (in the range of 10 to 30%) in the MazF-mediated expression of the STM reporters carrying an ACA site in each of the different five frame 0 locations (Fig. 2D, a to e). When we used an STM reporter without any ACA sites, MazF induction led to an approximately 50% increase of GFP expression; in contrast, when we used an STM reporter that included one ACA in frame 0 of the *gfp* sequence, MazF induction led to a decrease in GFP expression. Thus, we found that the presence of a single, in-frame ACA triplet caused an additive effect of an approximately 60 to 80% decrease in GFP expression (compare the first red bar with each of the other red bars in Fig. 2D). We understand that this decrease in STM-GFP expression was caused by MazF cleaving those individual ACA sites located in frame 0 of the *gfp* open reading frame (ORF) in the STM system. In order to confirm that these results were not obtained only because of NA induction of MazF, we studied also the effect of another stress condition. We used the serine analogue serine hydroxamate (SHX), leading to amino acid starvation and thereby to the synthesis of ppGpp that induces MazF (5, 10). As shown in Fig. S5 in the supplemental material, as in the case of NA, a reduction in the level of GFP was observed by using the STM-GFP reporters carrying an in-frame ACA in two different locations.

### Determination of MazF cleavage at in-frame ACA sites of a leaderless mRNA by the use of a molecular approach.

**FIG 3:**
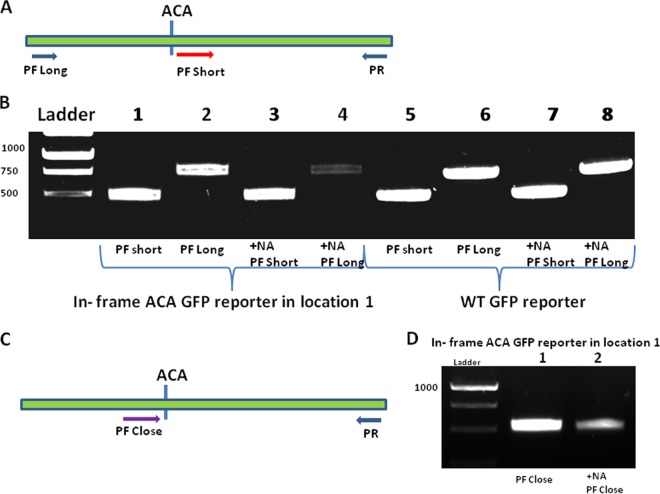
A molecular approach to study MazF cleavage at in-frame ACAs of the leaderless mRNA GFP reporters. (A) Illustration of the locations of primers designed for PCR amplification. The green line represents the STM-GFP reporter sequence including one in-frame ACA site. The “long primer forward” is marked “PF long” (blue arrow). The “short primer forward” is marked “PF short” (red arrow). The “reverse primer” is marked PR (blue arrow). (B) Agarose gel with samples of PCR products of GFP reporters (see Materials and Methods). Lane 1, the in-frame ACA GFP reporter with PCR amplification by a short primer. Lane 2, as in lane 1 but with PCR amplification by a long primer. Lane 3, as in lane 1 but with the addition of NA. Lane 4, as in lane 2 but with the addition of NA. Lanes 5 to 8, as in lanes 1 to 4 but with the WT GFP reporter. (C) As in panel A, but with a different forward primer; the close forward primer is marked PF close (purple arrow). (D) As in panel B but the GFP reporter has an in-frame ACA in location 1. Lane 1, PCR amplification with a close primer. Lane 2, as in lane 1 but with the addition of NA.

To confirm our indirect results using STM-GFP reporters, we developed a molecular method to determine MazF cleavage at in-frame ACA sites in a GFP leaderless mRNA. In this method, we extracted RNA from MazF-induced and uninduced MG1655 (WT) cells harboring either (i) an STM-GFP reporter with only one in-frame ACA site (location 1 in Fig. 2A) or (ii) the WT GFP reporter that carries 17 out-of-frame ACA sites. Using the extracted RNAs, we prepared corresponding cDNA samples which we amplified by a PCR. In this reaction, we determined the site of MazF cleavage using two different forward primers (PF Long and PF Short) and one reverse primer (PR) that we designed to start from the end of the GFP reporter sequence (Fig. 3A). We designed PF Long to start from the beginning of the sequence of the leaderless GFP reporters and PF Short to start directly after the in-frame ACA site that we generated in location 1 (Fig. 2A). We hypothesized that with the addition of NA to induce MazF expression, if MazF were to cleave at this in-frame ACA site of the leaderless STM-GFP mRNA, using PF Long would not lead to a reverse transcription-PCR (RT-PCR). Indeed, we observed almost no RT-PCR product when the MazF-induced sample was amplified by PF Long (Fig. 3B, lane 4). In contrast, this RT-PCR product was observed in the absence of NA, when no MazF expression was induced (Fig. 3B, lane 2). We suggest that the minimal amount of RT-PCR product seen in the *mazF*-induced sample using PF Long (Fig. 3B, lane 4) probably represents incomplete MazF cleavage at the in-frame ACA site of the reporters. To control for the quality and integrity of the RNA samples used after *mazF* induction, we used PF Short, designed to start immediately downstream from the in-frame ACA site. Using PF Short, we obtained similar amounts of RT-PCR products in MazF-induced (Fig. 3B, lane 3) and uninduced (Fig. 3B, lane 1) samples. Note that results similar to these obtained here (Fig. 3B) were also obtained while using a leaderless STM-GFP reporter harboring an in-frame ACA site in location 2 (see Fig. S6 in the supplemental material).

To support the evidence of MazF cleavage at the in-frame ACA cutting site, we designed an additional, close forward primer (PF Close) that ends immediately before the ACA site, upstream from the MazF cleavage site (Fig. 3C). Using the STM-GFP reporter with the in-frame ACA site in location 1 as with PF Long, with PF Close, we expected to observe a decrease in the PCR product after the addition of NA. Indeed, we found much less expression after the addition of NA (Fig. 3D, lane 2) than without the addition of NA (Fig. 3D, lane 1), indicating that when the endonuclease MazF was expressed, it cut at the in-frame ACA site. Note that when we used close primers that were designed right upstream of the ACA cutting site, we saw a decrease in band intensity. However, when we used the short forward primer designed right downstream of this cutting site (as described for Fig. 3A), we did not observe this decrease. Thus, we were able to zoom into the ACA cutting site and to confirm that indeed MazF cleaves at this in-frame ACA site.

Using this same technique, with the WT GFP reporter carrying 17 out-of-frame ACA sites (Fig. 1B), we also confirmed that out-of-frame ACAs were resistant to cleavage by MazF. Though (+1 frame) ACA sites were present, when we used PF Long, inducing MazF did not lead to a decrease in the amount of the PCR product obtained (Fig. 3B, compare lanes 6 and 8). Since all the ACA sites in the WT GFP reporter were in reading frame +1, we asked if ACA sites in a +2 frame would also be resistant to MazF cleavage. We constructed two different STM-GFP reporters carrying an ACA site in frame +2 (see Fig. S7 in the supplemental material). When we used PF Long, and after MazF induction, as we found for +1 frame ACAs, when we used +2 frame ACAs, we observed no reduction in the amount of the RT-PCR product obtained.

Together, our results confirmed that in the leaderless GFP mRNA, MazF did not cleave ACA when they were in the +1 frame or +2 frame but only when they were in frame 0.

### The EDF-like element in bS1 is involved in the MazF in-frame ACA cleavage of the STM-GFP reporter.

**FIG 4:**
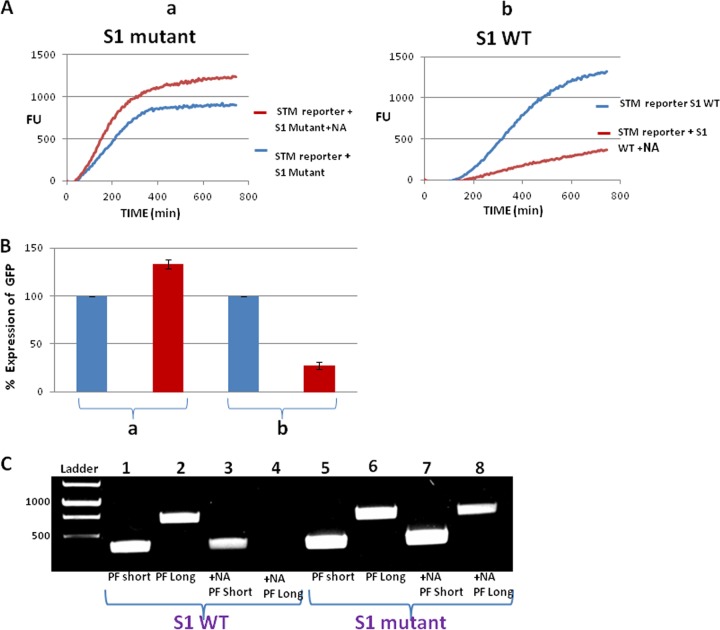
In the GFP-STM reporter, MazF-induced cleavage of in-frame ACA is dependent on EDF-like sequence in bS1. (A) (a) Effect of MazF induction on expression of the GFP-STM reporter in cells harboring a plasmid carrying the bS1 mutation W→A in the EDF-like sequence (mutation W444A). (b) As in panel a but in cells harboring a plasmid carrying WT bS1. These experiments were carried out as described in the legend to Fig. 2C; MazF expression was induced by the addition of NA. FU, fluorescence units. (B) Quantitative comparison of GFP expression in MazF-induced samples (red bars) versus uninduced samples (blue bars). Data were calculated as percentages from the results of panels Aa and Ab. In each assay, the value of 100% was given to the results for the untreated sample. (C) Agarose gel with samples of PCR products of the GFP-STM reporter carrying an in-frame ACA site (see Materials and Methods). Lanes 1 to 4, samples obtained from cells harboring a constitutive pACYC plasmid carrying the WT bS1. Lanes 5 to 8, sample obtained from cells harboring a constitutive pACYC plasmid carrying the bS1 mutation W→A in the EDF-like sequence (mutation W444A). Lanes 1 and 5, PCR amplification with a short primer; lanes 2 and 6, PCR amplification with a long primer; lanes 3 and 7, as in lanes 1 and 5 with the addition of NA; lanes 4 and 8, as in lanes 2 and 6 with the addition of NA.

The extracellular death factor (EDF), pentapeptide NNWNN (16), binds to MazF and is involved in its activity (17). We were surprised to find NNW, an EDF-like sequence, in the C-terminal domain of the ribosomal protein bS1. Previously, we showed that MazF binds to bS1 through the NNW sequence and that a W→A mutation in this sequence prevents the binding of MazF to bS1 (S. Kumar, B. Byrgazov, H. Engelberg-Kulka, and I. Moll, submitted for publication). Since here we found that cleavage by MazF was dependent on an in-frame ACA, we wondered if there might be a connection to MazF binding to bS1. We asked whether a W→A mutation in the EDF-like element of bS1 would prevent in-frame ACA MazF cleavage. First, we studied the effect of this mutation on the expression of the STM-GFP reporter, carrying an in-frame ACA site in location 1 (Fig. 2A). We found that in MG1655 WT cells harboring the multicopy pACYC plasmid carrying the bS1 gene (*rpsA*) with a W→A mutation, the expression of GFP was increased by about 35% after MazF induction (Fig 4Aa and 4Ba), in spite of the existence of an in-frame ACA site. Moreover, these results were unlike those of a similar experiment that we performed in which, instead of using the mutant bS1, we used the pACYC plasmid carrying the gene encoding WT bS1. In our experiments here, after MazF induction, GFP expression was severely reduced, by about 75% (Fig. 4A, b, and B, b). Thus, our quantitative analysis comparing results with bS1 WT and mutant bS1 revealed that, in the presence of the WT bS1, the induction of MazF led to an additive reduction of GFP expression of about 110% (Fig. 4B, compare red bars). We were able to support the role of the EDF-like element of bS1 in the reduction of the expression of the STM-GFP reporter after MazF induction by the results of additional experiments (see Fig. S8 in the supplemental material) in which we used four other in-frame ACA sites, generated each in a different location of the *gfp* sequence (Fig. 2A).

Finally, we confirmed the involvement of the EDF-like element of bS1 in the MazF in-frame ACA cleavage using the molecular method that we developed for determining ACA cleavage. Once again, we used the STM-GFP reporter with one in-frame ACA site in location 1 (Fig. 2A). Recall that, in this assay, if an ACA site were to be cleaved, the PCR product would not be obtained by the use of PF Long (Fig. 3). We observed the absence of this PCR product only in *mazF*-induced cells harboring a pACYC plasmid carrying the WT bS1 (Fig. 4C, lane 4). On the other hand, this PCR product was obtained without MazF induction (Fig. 4C, lane 2) and also in a MazF-induced sample in cells harboring a pACYC plasmid carrying the mutant bS1 (Fig. 4C, lane 8). This indicates that MazF does not cleave at the in-frame ACA site in the presence of a mutant bS1.

Our combined results suggest that, in STM, the EDF-like element in bS1 was involved in the MazF in-frame ACA cleavage, probably because through bS1 MazF becomes a part of the ribosome in the stress-induced translation machinery.

### In leaderless mRNAs to be translated by the STM system, all the ACA triplets are located out of frame.

**FIG 5:**
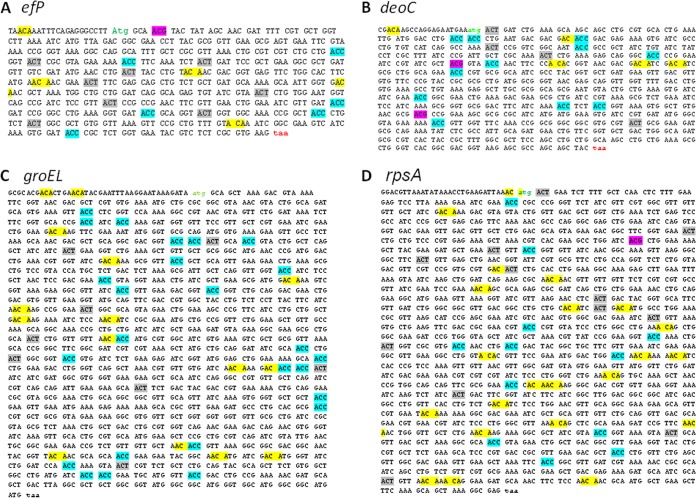
Locations of the synonym threonine codons in genes specifying MazF-induced regulon products. DNA sequences specifying EF-P (A), DeoC (B), GroEL (C), and bS1 (D). Synonym threonine codons are ACAs (highlighted in yellow), ACCs (in blue), ACT (in gray), and ACGs (in magenta).

Previously, we characterized MazF-induced small (less than 20-kDa) stress proteins that would be translated by STM (13). Among these were EF-P, DeoC, SoxS, RbfA, and AhpC. Here, we found that ACA sites located out of frame within leaderless mRNAs are resistant to MazF cleavage. So, we asked whether in the leaderless mRNAs the ACA triplets that specify these proteins are located in frame or out of frame. We found that all of the ACA triplets were situated out of frame in *efp* (Fig. 5A), *deoC* (Fig. 5B), and *soxS*, *rbfA*, and *ahpC* mRNAs (see Fig. S9A to C in the supplemental material). Note that in a few cases, as for *yfiD* and *yfbU*, we did find some ACAs located in frame (see Fig. S9D and E). We hypothesize that these represent minor examples, which might point to the existence of an additional mechanism(s) that may resist cleavage by MazF.

Moreover, we also observed another characteristic related to the genetic code. It is well known that ACA encodes the amino acid threonine. Since the presence of ACAs in the open reading frame of leaderless mRNA does not permit its translation under stress-induced MazF, threonine is encoded by the synonym codons ACC, ACU, and ACG, which are resistant to MazF cleavage (6) (Fig. 5A to D).

Furthermore, our recent studies, in which we identified the *E. coli* MazF leaderless regulon (15), permitted us here to identify mRNAs encoding larger proteins, including *rpsA*, which encodes the ribosomal bS1 protein (Fig. 5D) and *groEL* (Fig. 5C). As can be seen, *rpsA* has 21 out-of-frame ACA sites, and *groEL* has 13 out-of-frame ACA sites. Furthermore, in both cases, the synonym threonine codons ACC, ACU, and ACG are located in frame 0 of the open reading frame. The *rpsA* mRNA carries 12 ACCs, 12 ACUs, and one ACG (Fig. 5D), and the *groEL* mRNA carries 25 ACCs and 8 ACUs (Fig. 5C).

**FIG 6:**
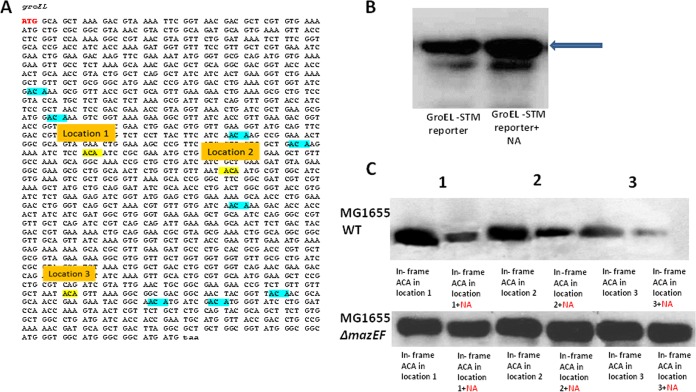
MazF-induced GroEL expression was reduced when ACAs were introduced in frame 0 of *groEL*. (A) Locations of three different in-frame ACA sites in the leaderless *groEL* sequence. Each ACA was inserted at a different location, generating only one ACA site in frame 0. The sites are numbered and highlighted in yellow. The different inserted ACAs were selected to be in place of and near the original out-of-frame ACA sites in the normal *groEL* sequence. (B) Western blot analysis (for details, see Materials and Methods) for the expression of a STM-GroEL reporter in MazF-induced MG1655 (WT) cells by NA. The blue arrow indicates the GroEL band. (C) Western blot assays for the expression of the three in-frame STM-GroEL reporters in MazF-induced MG1655 cells (upper panel) by NA. Bands are marked in 1, 2, and 3 corresponding to locations of the in-frame ACA *groEL* sequence used. Lower panel: same as in upper panel with the use of a Δ*mazEF* derivative of *E. coli* MG1655 cells.

We studied the case of *groEL* in depth, finding that when we induced MazF by adding NA, inserting even one in-frame ACA site into the *groEL* sequence caused a reduction in GroEL translation. As can be seen in Fig. 6, when we studied *groEL* expression after MazF induction by NA in the MG1655 (WT) strain, introducing an in-frame ACA site in any one of three different locations led to reduced expression of *groEL* (Fig. 6C). We observed no such reduction in the MG1655Δ*mazEF* derivative strain (Fig. 6C). In contrast to the decrease in mutant *groEL* expression that was observed in treated cells (Fig. 6C, upper row), when we used the construct containing the WT GroEL sequence, we obtained an increase in expression after MazF induction(Fig. 6B). This was due to the formation of a leaderless GroEL mRNA and because the WT GroEL does not carry any in-frame ACAs in its sequence.

## DISCUSSION

*E. coli* toxin MazF is well known to be a stress-induced (5, 10–12) endoribonuclease that cleaves at ACA sites (6, 7). The endoribonucleolytic activity of MazF has both a negative and a positive effect: it is an mRNA interferase that destroys mRNAs (6, 7), and it generates a stress-induced translation machinery—STM—composed of leaderless mRNAs and deficient ribosomes that selectively translate these mRNAs (14). Our inspection of the leaderless mRNA translated by the MazF-mediated STM revealed the existence of many ACA sites in these mRNAs. We observed that most of those ACA triplets are not located in frame 0, leading us to ask if a translation-dependent MazF ACA cleavage might occur in the leaderless mRNA. We found that this is indeed the case. We have characterized this translation-dependent MazF cleavage, finding that in the STM system, only in-frame ACA sites are cleaved, while out-of frame ACA sites are resistant to the cleavage (Fig. 1D and 2). We were able to support this important conclusion using different experimental systems. We compared the effects of MazF induction on the expression of STM-GFP reporters carrying ACA sites at different locations in the open reading frame of GFP (Fig. 1D and 2). We confirmed this in-frame dependency by using a molecular approach showing MazF cleavage at a specific in-frame ACA site in the GFP leaderless mRNA (Fig. 3).

**FIG 7:**
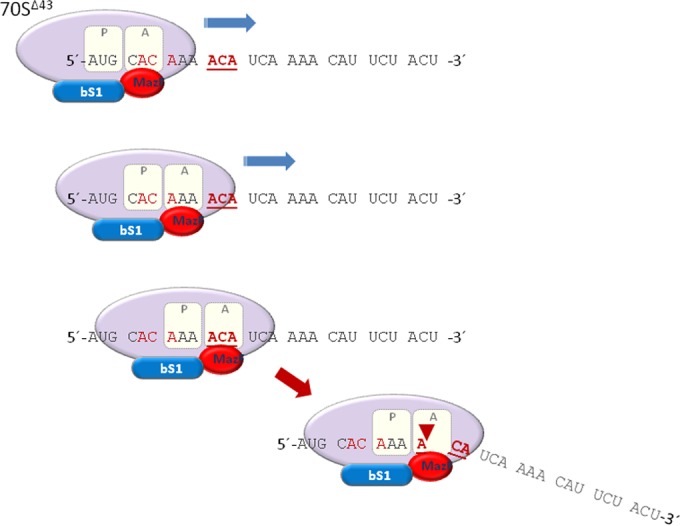
Model for frame-dependent MazF cleavage in the STM system under stress conditions. MazF becomes a part of the stress-induced ribosome through its attachment to the bS1 ribosomal protein in the 30S ribosomal subunit. The translation is performed according to the open reading frame. Movement of the ribosome is illustrated by the arrows. When the ribosome reaches an out-of-frame ACA (marked in red), translation is not interrupted. When it reaches an in-frame ACA (marked in bold red), MazF cleaves the mRNA, and translation is prevented.

Previously, we demonstrated that the pentapeptide NNWNN (EDF) enhances MazF activity on the one hand (16, 17) and that MazF also binds to the EDF-like element NNW in the ribosomal protein bS1 (Kumar et al., submitted). These results led us to ask, here, whether MazF in-frame ACA cleavage is related to the binding of MazF to the EDF-like element of bS1. Indeed, we found that a W→A mutation prevented a frame-dependent MazF cleavage at in-frame ACA sites (Fig. 4A), suggesting a model for frame-dependent MazF cleavage (Fig. 7). In our model, stressful conditions result in the induction of MazF and the generation of the STM system. MazF binds to STM ribosomal protein bS1 and becomes a part of the ribosome that proceeds along the mRNA. In this way, the process of mRNA translation and ACA in-frame cleavage are coupled in STM.

It should be emphasized that the frame-dependent ACA cleavage by MazF is unique to the STM system. In addition, MazF targets ACAs that are not connected to this phenomenon. These include (i) mRNAs translated by canonical ribosomes, by which MazF acts as an RNA interferase (7); (ii) removal of 43 nucleotides from the 3′ 16S rRNA when located in the ribosome, thereby producing the deficient ribosomes of STM (14); and (iii) cleavage of ACA sites located upstream of AUG initiation codons, thereby producing leaderless mRNAs (14) or processed mRNAs (15).

It was previously reported that 99% of *E. coli* mRNAs contain ACA triplets, but this was without relating to their reading frame (18). We have studied the distribution of *E. coli* mRNA ACA triplets in relation to the open reading frames. Our preliminary studies have revealed that, in contrast to STM, the *E. coli* mRNAs to be translated by the canonical ribosomes preferentially carry ACA sites in their open reading frames; 70% of the tested canonical mRNAs carry an ACA triplet in their open reading frames (see Fig. S10 in the supplemental material). This observation supports our conclusion that the novel translation-dependent stress-induced MazF-mediated ACA cleavage mechanism described here is specific to the STM system that is generated under stress. Moreover, our results have also shown that the STM system causes a bias in the reading of the genetic code. In the so-far-described products of the STM system, the synonym threonine codons ACC, ACU, and ACG are used instead of ACA (Fig. 5). Thus, stressful conditions may cause a bias in the way in which the genetic code is read. In fact, if stress also induces frameshifting in the canonical mRNAs, the translation-dependent MazF ACA cleavage operating in the STM system may serve as a mechanism that prevents frameshifting and, in that way, may prevent cleavage of out-of-frame ACAs. Of course, the novel translation-dependent stress-induced MazF-mediated ACA cleavage mechanism and the bias in the reading of the genetic code described here are so far restricted to *E. coli,* in which *E. coli* MazF recognizes and cleaves the ACA triplet. For most of the other MazF proteins described so far, as in *Bacillus subtilis* (19), *Staphylococcus aureus* (20), and *Mycobacterium tuberculosis* (21), the MazF recognition site is larger than a triplet. However, even one of the *Mycobacterium tuberculosis* MazF proteins, MazF-mt1, cleaves the triplet UAC (22). In addition, the recently discovered MazF of *Nitrosomonas europaea* cleaves a triplet, AAU (23). It is also possible that microorganisms in which the so-far-described recognition of their MazFs is larger than a triplet carry in addition another MazF(s) recognizing a triplet.

Further studies will reveal if the described bias in the reading of the genetic code is specific only for *E. coli* or if it may take place in other prokaryotes, or indeed in some eukaryotes. We already know that in the eukaryote jellyfish, the mRNA of the gene coding for GFP carries 17 ACAs, all of which are located out of frame (Fig. 1B). Also, in the open reading frame, the jellyfish GFP carries the threonine synonym codons, 17 ACCs, and one ACU (see Fig. S9F in the supplemental material). Since both this eukaryotic organism and the prokaryote *E. coli* appear to have the same bias in the reading of the genetic code, our results may show the beginning of a new way of reading the genetic code under stress. Our studies gain particular interest due to recent attempts to rewrite the genetic code by eliminating several synonym codons (24).

## MATERIALS AND METHODS

Primers for cloning were purchased from Integrated DNA Technologies, Inc. (IDT; Hudson, NH). Black 96-well plates were purchased from Nunc (Thermo Fisher Scientiﬁc, Denmark). Other materials and suppliers were as follows: RNeasy minikit, Qiagen, Hilden, Germany; PrimeSTAR HS DNA polymerase, TaKaRa Bio Inc., Otsu, Shiga, Japan; ProtoScript Moloney murine leukemia virus (MMuLV) first-strand cDNA synthesis kit, New England BioLabs; antibody AB-AB90522 (anti-*groEL* antibody) and AB-AB16284 (donkey anti-rabbit immunoglobulin [IgG] heavy and light chain [H&L]), Abcam, Inc., United Kingdom.

### Bacterial strains and plasmids.

We used the following set of *E. coli* strains: MG1655 (WT) (25) and its Δ*mazEF* derivative (26). Plasmid pUH-C, a pUH21-2 derivative (kindly provided by H. Bujard, University of Heidelberg), was derived from pBR322 (27). pACYC-rpsA-flag WT harbors the WT bS1 sequence. pACYC-rpsA-flag w444a carries the bS1 mutation sequence in the EDF-like element (Kumar et al., submitted). pKK-233-3 (constitutive plasmid, Amp^r^) carries the leaderless GroEL reporters as described below.

### Construction of the STM-GFP reporters.

The GFP variant that we used in reporter construction is derived from the Emerald-GFP (EmGFP; pRSET-*em-gfp*; Invitrogen, CA, USA), which is the brightest available GFP variant with very fast folding kinetics and distinct excitation and emission peaks of 487 nm and 509 nm, respectively (28). All STM-GFP reporters were constructed on the pUH-C plasmid. They carry a *gfp* sequence in which the first ATG is preceded by AC, generating an ACATG sequence that enables MazF cutting at the ACA, creating a leaderless mRNA of GFP. In addition, the ACATG is preceded by a stem-loop structure (created by using the sequence ggccgcAgcggcc) and thereby prevents Shine-Dalgarno sequence recognition.

Four kinds of reporters which differ in the *gfp* sequence downstream of the start codon were constructed as follows.

### (i) STM-GFP reporter with no ACA sites in the *gfp* sequence.

All ACA sites (all located in reading frame +1) were exchanged for ATA sequences (Fig. 1A), thus eliminating all MazF cleavage sites while maintaining the protein coding sequence. The Em*gfp*ΔACA gene including a 5′ untranscribed region (UTR) was synthesized *in vitro* by GeneArt (Regensburg, Germany; pMA-T_ΔACA-EmGFP_BamHI). The plasmid pUH-C_ΔACA-EmGFP was generated by amplification of the ΔACA-Em*gfp* gene with the forward primer IM_R13 and reverse primer IM_N9. The pUH-C_ΔACA-EmGFP 5′ UTR sequence is TTGACTTGTGAGCGGATAACAATGATACTTAGATTCAAGAATTCGGCCGCAGCGGCCAA**ACATG**, where the AUG start codon of Em*gfp* is shown in bold, the stem-loop structure in the conditional leaderless reporter is underlined, and the ACA site is highlighted in bold. The primers used for reporter construction are IM_R13-PF, ATAGAATTCGGCCGCAGCGGCCAAACATGGTGAGCAAGGGCGAGGAGCTGTTCA, and IM_N9-PR, TTACCCGGGTTACTGCAGTTACTTATACAGCTCGTC.

### (ii) STM-GFP reporter with the sequence of the WT *gfp* harboring 17 ACA sites.

We replaced the *gfp* sequence with no ACA sites in the STM-GFP reporter (see paragraph above) with the WT *gfp* sequence (Fig. 1A). This was created by cutting and ligating using the BamHI and EcoRI restriction sites in the pUH-C plasmid.

### (iii) STM-GFP reporters with one ACA site in frame 0 in different locations of the *gfp* sequence (Fig. 2A).

We constructed 5 STM-GFP reporters with one ACA site in frame 0 in different locations of the *gfp* sequence by the use of the following oligonucleotide primers: for ACA in location 1, 5′-TACCCCGACCATACAAAGCAGCAC-3′ for sense sequence and 3′-GTGCTGCTTTGTATGGTCGGGGTA-5′ for antisense sequence; for ACA in location 2, 5′-GACGGCAACTATACAACCCGCGCC-3′ for sense sequence and 3′-GGCGCGGGTTGTATAGTTGCCGTC-5′ for antisense sequence; for ACA in location 3, 5′-TTCGAGGGCGATACACTGGTGAAC-3′ for sense sequence and 3′-GTTCACCAGTGTATCGCCCTCGAA-5′ for antisense sequence; for ACA in location 4, 5′-GAGTATAACTATACAAGCCATAAG-3′ for sense sequence and 3′-CTTATGGCTTGTATAGTTATACTC-5′ for antisense sequence; for ACA in location 5, 5′-TACCAGCAGAATACACCCATCGGC-3′ for sense sequence and 3′-GCCGATGGGTGTATTCTGCTGGTA-5′ for antisense sequence.

PCR programs were carried out in which only a few cycles of annealing were performed to prevent extra mutations (5 cycles, first annealing stage; 10 cycles, second annealing stage). This procedure created unmethylated newly mutated synthesized plasmid. Finally, DpnI enzyme was added to cut the methylated but not unmethylated DNA, thereby eliminating the original plasmid and leaving only the newly mutated synthesized plasmid. All generated mutations were confirmed by sequencing.

### (iv) STM-GFP reporters with one ACA site in frame +2 in different locations of the *gfp* sequence.

We constructed 5 STM-GFP reportes with one ACA site in frame +2 in different locations of the *gfp* sequence by the use of the following oligonucleotides: for ACA in location 1, 5′-TACCCCGACCAACAGAAGCAGCAC-3′ for sense sequence and 3′-GTGCTGCTTCTGTTGGTCGGGGTA-5′ for antisense sequence; for ACA in location 2, 5′-GGCAACTATAAACACCGCGCCGAG-3′ for sense sequence and 3′-CTCGGCGCGGTGTTTATAGTTGCC-5′ for antisense sequence; for ACA in location 3, 5′-TTCGAGGGCGAACACCTGGTGAAC-3′ for sense sequence and 3′-GTTCACCAGGTGTTCGCCCTCGAA-5′ for antisense sequence; for ACA in location 4, 5′-AACTATAAACACCATAAGGTCTAT-3′ for sense sequence and 3′-ATAGACCTTATGGTGTTTATAGTT-5′ for antisense sequence; for ACA in location 5, 5′-CAGCAGAAACACCCCATCGGC-3′ for sense sequence and 3′-GCCGATGGGGTGTTTCTGCTG-5′ for antisense sequence.

The PCR program was done as described above.

### Construction of a canonical GFP reporter.

The plasmid pMS2_112_pMAT-dACA-EmGFP_BamHI.gbk comprising the EmGFPΔACA gene downstream of a canonical 5′ UTR was purchased from GeneArt (Regensburg, Germany) and then cloned into the pUH-C plasmid. The 5′ UTR sequence of this construct is as follows (the SD sequence is indicated in italics): TTGACTTGTGAGCGGATAACAATGATACTTAGATTCAGAATTCTCGCCAGGGGTGCTCGGCATAAGCCGAAGATATCGGTAGAGTTAATATTGAGCAGATCCCCCGGTGAAGGATTTAACCGTGTTATCTCGTTGGAGATATTCATGGCGTATTTTGGATCCT*AACGAGG*CGCAAAAA**ATG**.

### Construction of the STM-GroEL reporters.

All STM-GroEL reporters were constructed on the pKK-233-3 constitutive plasmid. Two kinds of reporters were designed by us.

### (i) Construction of WT STM-GroEL reporter.

The construct carries a *groEL* sequence in which the first ATG is preceded by AC, generating an ACATG sequence that enables MazF cutting at the ACA, creating a leaderless mRNA of GFP. In addition, the ACATG is preceded by a stem-loop structure (created by using the sequence ggccgcAgcggcc), thereby preventing the Shine-Dalgarno sequence recognition (similar to the STM-*gfp* reporter construction described above). Here, all ACA sites in the sequence remained unchanged. The construct was inserted by cloning into the pKK-233-3 constitutive plasmid using SmaI and EcoRI restriction enzymes. Primers used for cloning were PF-*groEL*, tGAATTCggccgcAgcggccAAACatgGCAGCTAAAGACGTAAAA, and PR-*groEL*, tcccgggttaATGGTGATGGTGATGGTGCATCATGCCGCCCATGCCACCCATGCC (lowercase letters indicate the restriction enzyme recognition site).

### (ii) Construction of 3 STM-GroEL reporters carrying one in-frame ACA site at a different location on the *groEL* sequence.

Here, we used the leaderless *groEL* mRNA as a platform. ACA insertions were performed by the use of the following oligonucleotide primers: for ACA in location 1, 5′-AAAATCTCCACAATCCGCGAAATG-3′ for sense sequence and 3′-CATTTCGCGGATTGTGGAGATTTT-5′ for antisense sequence; for ACA in location 2, 5′-CTGGTTGTTAATACAATGCGTGGC-3′ for sense sequence and 3′-GCCACGCATTGTATTAACAACCAG-5′ for antisense sequence; for ACA in location 3, 5′-GTTGTTGCTAATACAGTTAAAGGC-3′ for sense sequence and 3′-GCCTTTAACTGTATTAGCAACAAC-5′ for antisense sequence.

### Growth conditions and assays for measuring GFP expression.

*E. coli* MG1655 (WT) and its Δ*mazEF* derivative were transformed with plasmid pUH-C harboring each of the different GFP-STM reporters. The transformed bacteria were grown in 10 ml M9 medium containing 0.2% glucose and in the presence of ampicillin (100 μg/ml) at 37°C with shaking (250 rpm) until reaching an optical density at 600 nm (OD_600_) of 0.4 to 0.5. Triplicate samples of WT and Δ*mazEF* strains were applied to wells in a black 96-well plate and were untreated or treated with nalidixic acid (NA) (100 μg/ml) to induce MazF activity. In the assay described in the legend to Fig. S5 in the supplemental material, samples were treated with serine hydroxamate (SHX) (60 μg/ml) to induce MazF. GFP levels were detected with a FLUOstar spectrophotometer. We measured fluorescence using a 485- ± 15-nm excitation filter and a 520- ± 15-nm emission filter, for 150 times at intervals of 300 s (total time of experiment, 750 min). The temperature in the device was kept at 37°C. The GFP fluorophore was excited with 1,000 units lamp energy, and the fluorescence in each well was measured for 5 s (FLUOstar Galaxy; BMG Labtechnologies). In assays using plasmids pACYC-rpsA-flag WT and pACYC-rpsA-flag w444a, the same procedure was carried out with the addition of chloramphenicol (25 μg/ml) to the M9 medium.

### Molecular approach to determine MazF cleavage at in-frame ACA sites of a leaderless GFP mRNA.

*E. coli* MG1655 (WT) cells were transformed with plasmid pUH-C harboring one of the GFP-STM reporters. Cells were grown until reaching an OD_600_ of 0.4 to 0.5 as described above and treated with NA (100 μg/ml) for 4 h. For RNA extraction, we used the RNeasy minikit from Qiagen as directed by the manufacturer. In order to discard DNA residues, we also used the RNase-free DNase set from Qiagen. From the RNA samples, cDNA samples were prepared by the use of the ProtoScript MMuLV first-strand cDNA synthesis kit (New England BioLabs). Synthesis was done by using the general primers provided with the kit. Next, in order to amplify the cDNA samples obtained, we performed PCRs using 3 different forward primers (PF) and one reverse primer (PR). PCR was set to only 15 cycles of annealing in order to be able to compare the quantities of the PCR products obtained.

### Primers used for PCR amplification.

For sample containing the STM-GFP reporter with one in-frame ACA site in location 1, the normal GFP reporter, and the reporter with one ACA site in frame +2 in location 1, primers were PF Long, 5′-AAGGGCGAGGAGCTGTTC-3′; PF Short, 5′-AAGCAGCACGACTTCTTCAAG-3′; PF Close, 5′-GCCCGCTACCCCGACCAT-3′; and PR, 3′-ATACAGCTCGTCCATGCCG-5′. For sample containing the STM-GFP reporter with one in-frame ACA site in location 2, primers were PF Long, 5′-ATGGTGAGCAAGGGCGAGGA-3′; PF Short, 5′-ACCCTGGTGAACCGCATC-3′; PF Close, 5′-TTCAAGGACGACGGCAACTAT-3′; and PR, 3′-GATCCCGGCGGCGGTCAC-5′. For sample containing the STM-GFP reporter with an ACA site in frame +2 in location 2, the primer was PF Short, 5′-CGCGCCGAGGTGAAGTTC-3′. The other primers were as described for samples containing the STM-GFP reporter with one in-frame ACA site in location 1, the normal GFP reporter, and the reporter with one ACA site in frame +2 in location 1. Finally, samples were run on a 1% agarose gel containing ethidium bromide (Fig. 3A and B and 4C).

### Western blot assay for GroEL expression.

*E. coli* MG1655 (WT) and its Δ*mazEF* derivative were transformed with plasmid pKK-233-3 harboring each of the different STM-GroEL reporters. The transformed bacteria were grown in 10 ml M9 medium containing 0.2% glucose and in the presence of ampicillin (100 μg/ml) at 37°C with shaking (250 rpm) until they reached an OD_600_ of 0.4 to 0.5. Then, samples were untreated or treated with NA (100 μg/ml) for 13 h. Proteins were extracted by cell lysis using lysozyme (20 mg/ml) and run on a 12% polyacrylamide-SDS gel. Polyvinylidene difluoride (PVDF) membrane transfer was done by semidry blotting with Fastblot B44 (Biometra GmbH, Göttingen, Germany). The primary antibody used was AB-AB90522 (anti-GroEL antibody). The secondary antibody used was AB-AB16284 (donkey anti-rabbit IgG H&L)–horseradish peroxidase (HRP). ECL solution (Biological Industries, Israel) was added to the PVDF membrane for HRP enzymatic reaction. Finally, the membrane was exposed to Fuji medical X-ray film.

## SUPPLEMENTAL MATERIAL

Figure S1  Effect of MazF induction on canonical GFP expression. (A) Illustration of the canonical *gfp* sequence construct containing the Shine-Dalgarno sequence. No ACA sites are present upstream of the AUG initiation codon. (B) GFP expression in *E. coli* strain MG1655 transformed with plasmid pUH-C carrying a canonical GFP reporter. FU, fluorescence units. Download Figure S1, TIF file, 0.1 MB

Figure S2  Effect of NA on growth of *E. coli* MG1G55 (WT) cells. *E. coli* MG1G55 (WT) cells were grown in 10 ml M9 medium containing 0.2% glucose and at 37°C with shaking (250 rpm), until they reached an OD_600_ of 0.5. Then, cells were untreated (blue line) or treated with nalidixic acid (100 μg/ml) to induce MazF activity (red line). OD_600_ measurements were taken every 60 min. Download Figure S2, TIF file, 0.1 MB

Figure S3  Expression of the WT GFP reporter in MG1655 Δ*mazEF* cells. The experiment was done as described in the legend to Fig. 1D with the use of Δ*mazEF* cells of *E. coli* MG1655. Download Figure S3, TIF file, 0.1 MB

Figure S4  The expression of the GFP-STM reporter carrying ACA at different frame 0 sites is not affected in a Δ*mazEF* derivative of *E. coli* MG1655 (WT). The experiment was done as described in the legend to Fig. 2 with the use of Δ*mazEF* cells of *E. coli* MG1655. Download Figure S4, TIF file, 0.1 MB

Figure S5  MazF induced by serine hydroxamate (SHX) led to reduced GFP expression in STM-GFP reporters carrying an ACA site in frame 0. Experiments were done as described in the legend to Fig. 2. The blue bars represent untreated samples. Red bars represent samples treated with serine hydroxamate (SHX) (60 μg/ml) to induce MazF. (A) Quantitative comparison of GFP expression levels (percent). Columns 1 and 2, *E. coli* MG1655 (WT) cells harboring a GFP-STM reporter with no ACA sites; columns 3 and 4, *E. coli* MG1655 (WT) cells harboring a GFP-STM reporter with one in-frame ACA site in location 1 (location is described in the legend to Fig. 2A); columns 5 and 6, same as in columns 3 and 4 but in *ΔmazEF* derivative of *E. coli* MG1655 (WT) cells. (B) Same as in panel A with the use of a GFP-STM reporter with one in-frame ACA site in location 2. Download Figure S5, TIF file, 0.1 MB

Figure S6  A molecular approach to study the MazF cleavage at in-frame ACA sites of the leaderless mRNA GFP reporter carrying an ACA site at location 2. The experiment was done as described in the legend to Fig. 3; the location of ACA in frame 0 on the GFP-STM reporter was changed to location 2 (Fig. 2A). Download Figure S6, TIF file, 0.1 MB

Figure S7  A molecular approach to study the MazF cleavage at ACA sites in frame +2 of the leaderless mRNA GFP reporters. The experiment was done as described in the legend to Fig. 3 but with two different GFP-STM reporters each carrying an ACA site in frame +2. Download Figure S7, TIF file, 0.1 MB

Figure S8  The MazF-induced cleavage of an in-frame ACA site (in all 5 different locations) in the GFP-STM reporter is dependent on the EDF-like sequence in bS1. The experiment was done as described in the legend to Fig. 4. All 5 different GFP-STM in-frame ACA reporters were used. (A) Quantitative comparison of GFP expression in MazF-induced samples (red bars) versus uninduced samples (blue bars) in cells harboring the pUH-C plasmid carrying the gene coding for a mutant bS1. (B) As in panel A with a WT bS1. Download Figure S8, TIF file, 0.1 MB

Figure S9  Locations of threonine synonyms ACA, ACC, ACG, and ACT in examples of the genes specifying MazF-induced regulon and in the GFP sequence. DNA sequences are presented: *soxS* (A), *rbfA* (B), *ahpC* (C), *yfiD* (D), *yfbU* (E), and *gfp* (F). Out-of-frame ACA sites are highlighted in yellow, in-frame ACAs are highlighted in red, ACCs are highlighted in blue, ACGs are highlighted in magenta, and ACTs are highlighted in gray. Download Figure S9, TIF file, 0.2 MB

Figure S10  Distribution of ACA triplets in relation to the open reading frames in *E. coli* canonical mRNAs. The sequences upstream of all *E. coli* MG1655 genes were scanned. A total of 2,807 genes (circled in blue) were found to have a coding-free region of at least 30 nucleotides long upstream of their translation start site. These sequences were extended upstream up to 100 nucleotides long (when noncoding) and scanned for ACA. Five hundred seventy-five sequences (circled in green) of the chosen 2,807 (20% of the genes) had no ACA upstream of the initiation codon AUG. Therefore, these genes are considered to be canonical mRNAs rather than MazF-induced leaderless mRNAs. Circled in red is a subgroup of the canonical mRNAs (circled in green) that carry an ACA triplet in their open reading frames (70% of the genes). Download Figure S10, TIF file, 0.02 MB
