# Supplementary figures and images for: A Stress-Induced Bias in the Reading of the Genetic Code in Escherichia coli
Source: mBio. 2016 Nov 15;7(6):e01855-16. doi: 10.1128/mBio.01855-16 (PMC5111409; doi:10.1128/mBio.01855-16)

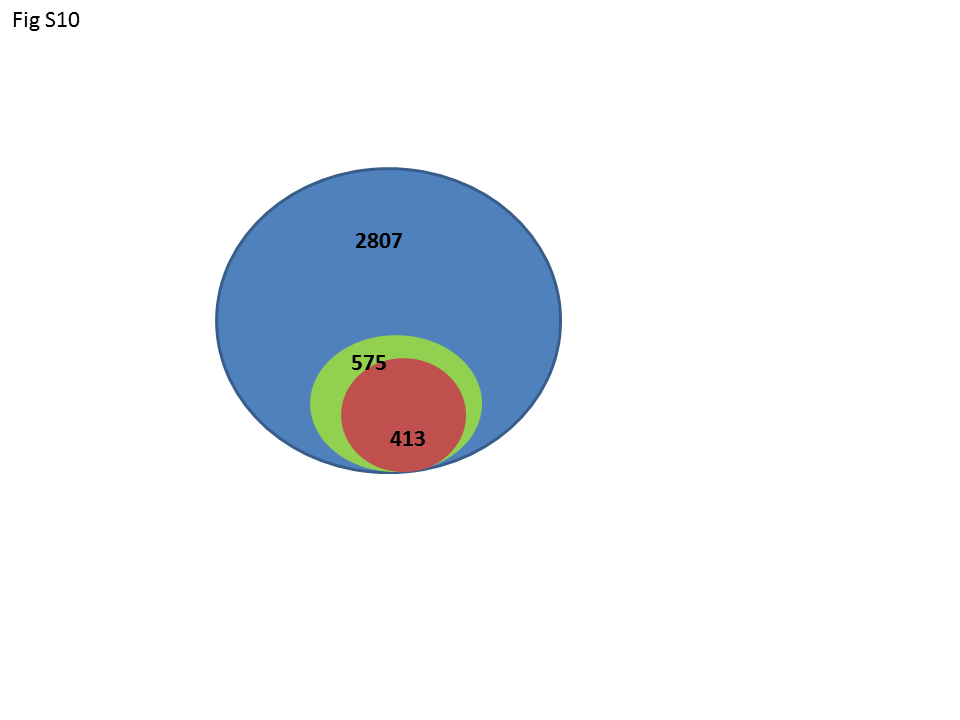

Supplement: Figure S1 — Effect of MazF induction on canonical GFP expression. (A) Illustration of the canonical gfp sequence construct containing the Shine-Dalgarno sequence. No ACA sites are present upstream of the AUG initiation codon. (B) GFP expression in E. coli strain MG1655 transformed with plasmid pUH-C carrying a canonical GFP reporter. FU, fluorescence units. Download [file mbo006163073sf10.tif]

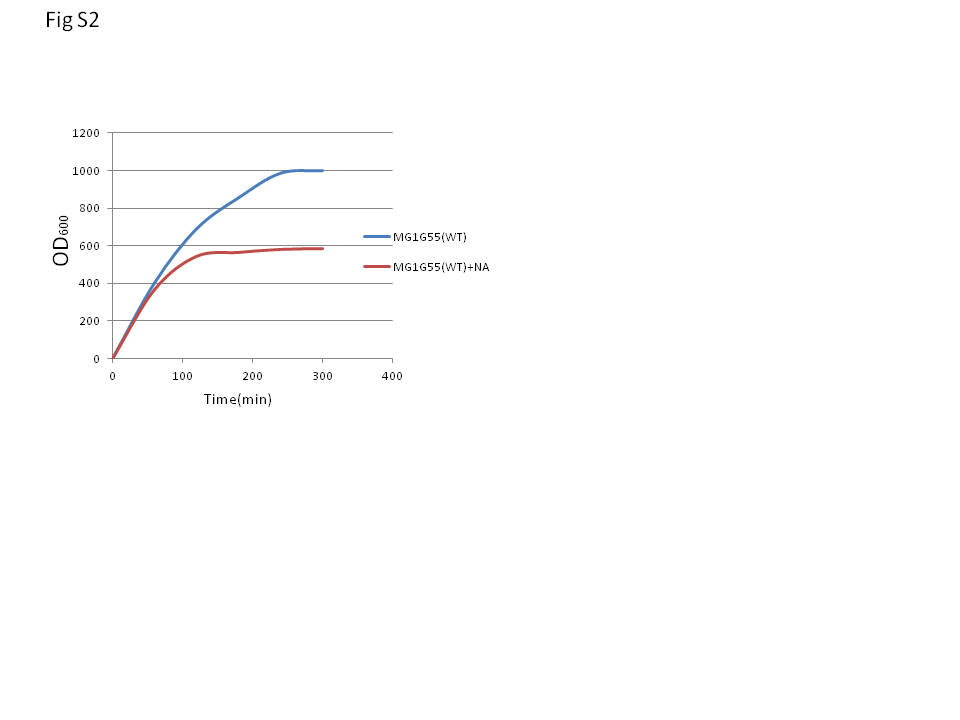

Supplement: Figure S2 — Effect of NA on growth of E. coli MG1G55 (WT) cells. E. coli MG1G55 (WT) cells were grown in 10 ml M9 medium containing 0.2% glucose and at 37°C with shaking (250 rpm), until they reached an OD600 of 0.5. Then, cells were untreated (blue line) or treated with nalidixic acid (100 μg/ml) to induce MazF activity (red line). OD600 measurements were taken every 60 min. Download [file mbo006163073sf2.tif]

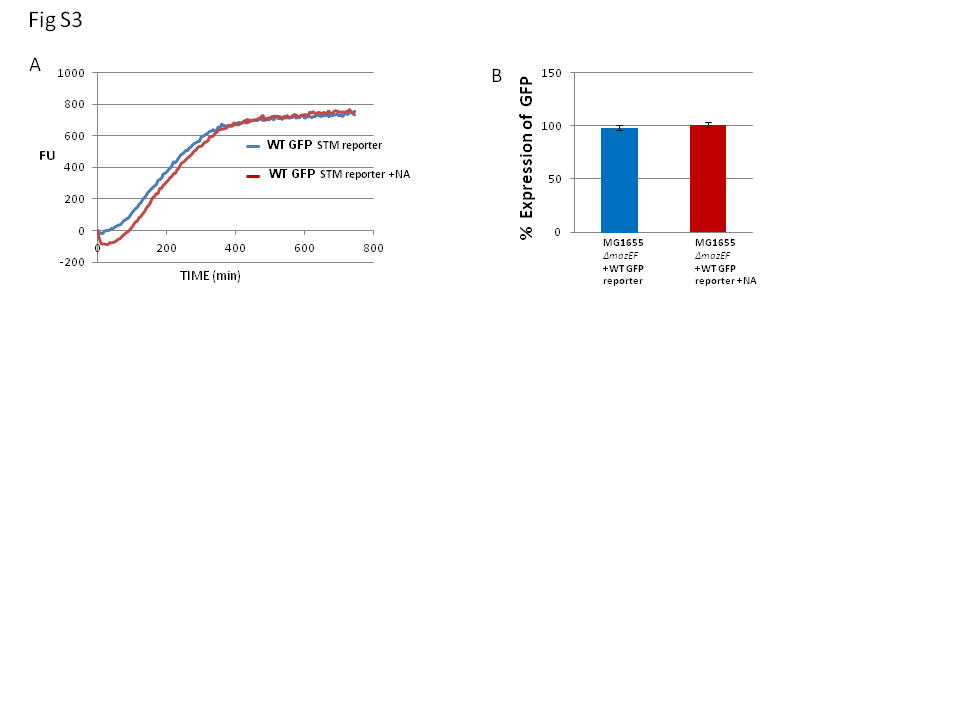

Supplement: Figure S3 — Expression of the WT GFP reporter in MG1655 ΔmazEF cells. The experiment was done as described in the legend to Fig. 1D with the use of ΔmazEF cells of E. coli MG1655. Download [file mbo006163073sf3.tif]

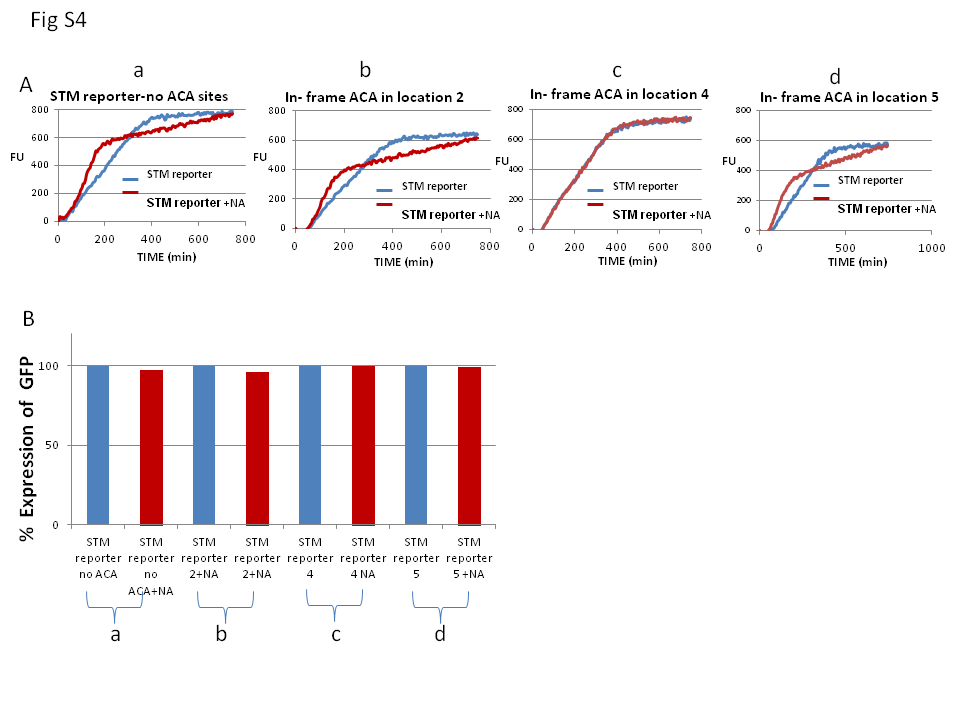

Supplement: Figure S4 — The expression of the GFP-STM reporter carrying ACA at different frame 0 sites is not affected in a ΔmazEF derivative of E. coli MG1655 (WT). The experiment was done as described in the legend to Fig. 2 with the use of ΔmazEF cells of E. coli MG1655. Download [file mbo006163073sf4.tif]

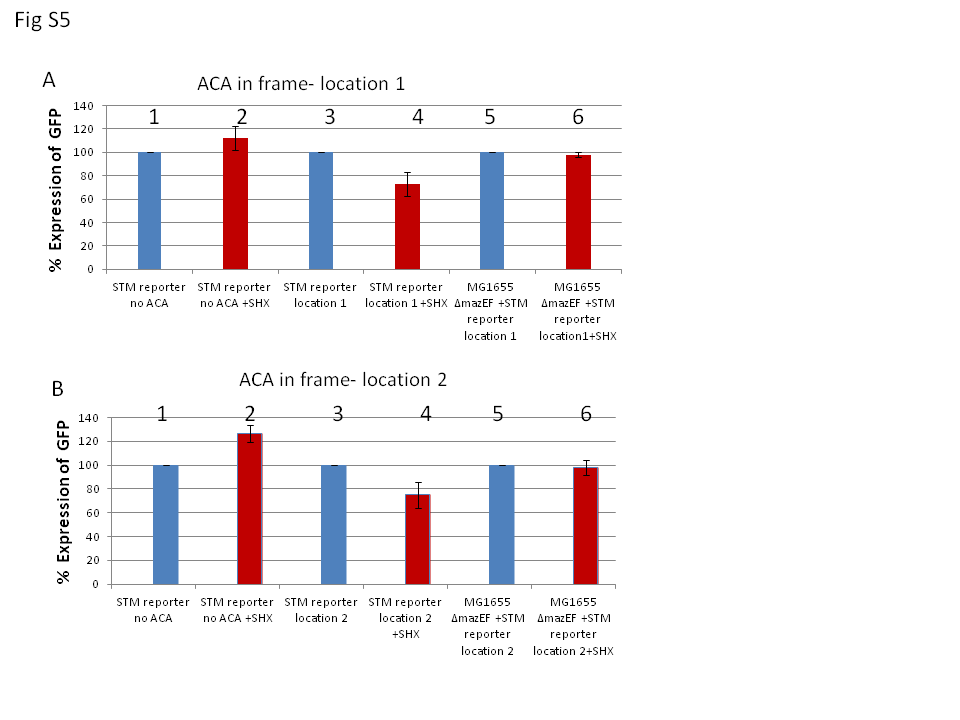

Supplement: Figure S5 — MazF induced by serine hydroxamate (SHX) led to reduced GFP expression in STM-GFP reporters carrying an ACA site in frame 0. Experiments were done as described in the legend to Fig. 2. The blue bars represent untreated samples. Red bars represent samples treated with serine hydroxamate (SHX) (60 μg/ml) to induce MazF. (A) Quantitative comparison of GFP expression levels (percent). Columns 1 and 2, E. coli MG1655 (WT) cells harboring a GFP-STM reporter with no ACA sites; columns 3 and 4, E. coli MG1655 (WT) cells harboring a GFP-STM reporter with one in-frame ACA site in location 1 (location is described in the legend to Fig. 2A); columns 5 and 6, same as in columns 3 and 4 but in ΔmazEF derivative of E. coli MG1655 (WT) cells. (B) Same as in panel A with the use of a GFP-STM reporter with one in-frame ACA site in location 2. Download [file mbo006163073sf5.tif]

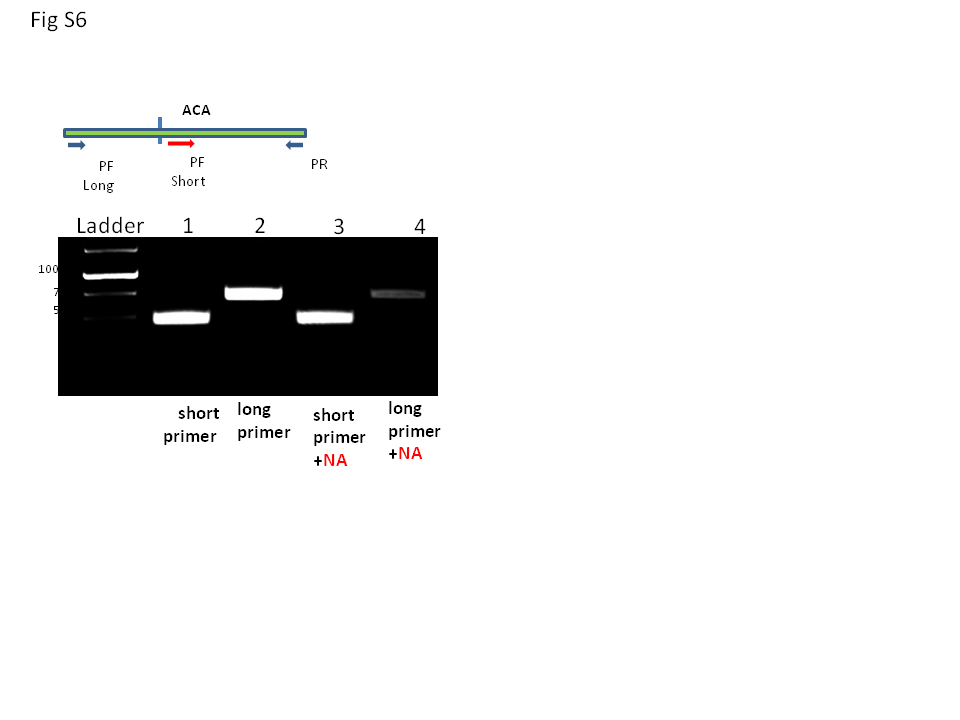

Supplement: Figure S6 — A molecular approach to study the MazF cleavage at in-frame ACA sites of the leaderless mRNA GFP reporter carrying an ACA site at location 2. The experiment was done as described in the legend to Fig. 3; the location of ACA in frame 0 on the GFP-STM reporter was changed to location 2 (Fig. 2A). Download [file mbo006163073sf6.tif]

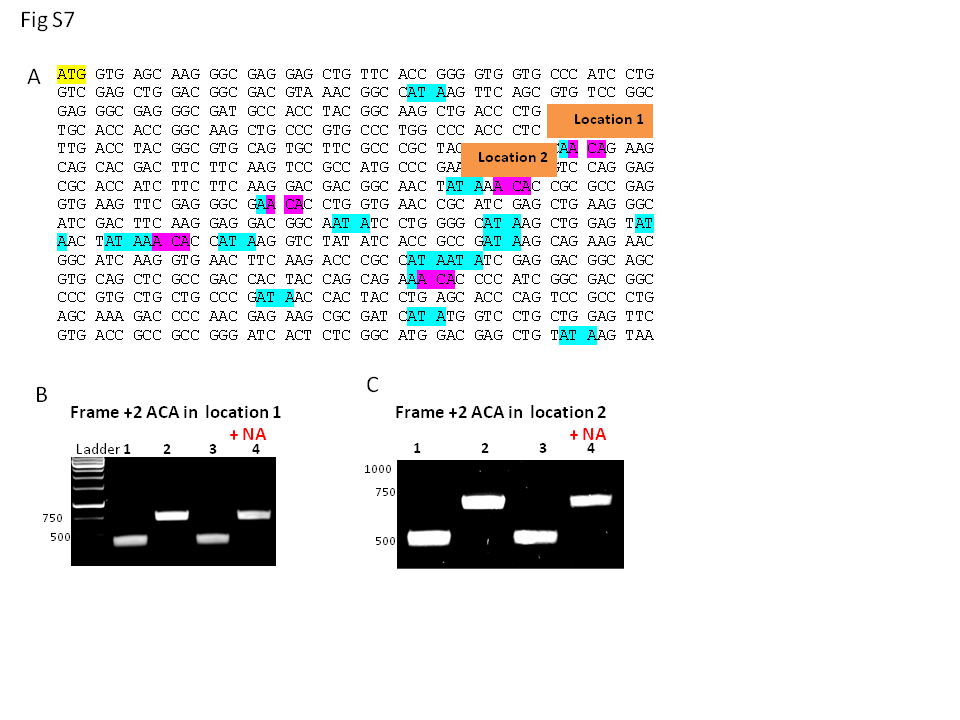

Supplement: Figure S7 — A molecular approach to study the MazF cleavage at ACA sites in frame +2 of the leaderless mRNA GFP reporters. The experiment was done as described in the legend to Fig. 3 but with two different GFP-STM reporters each carrying an ACA site in frame +2. Download [file mbo006163073sf7.tif]

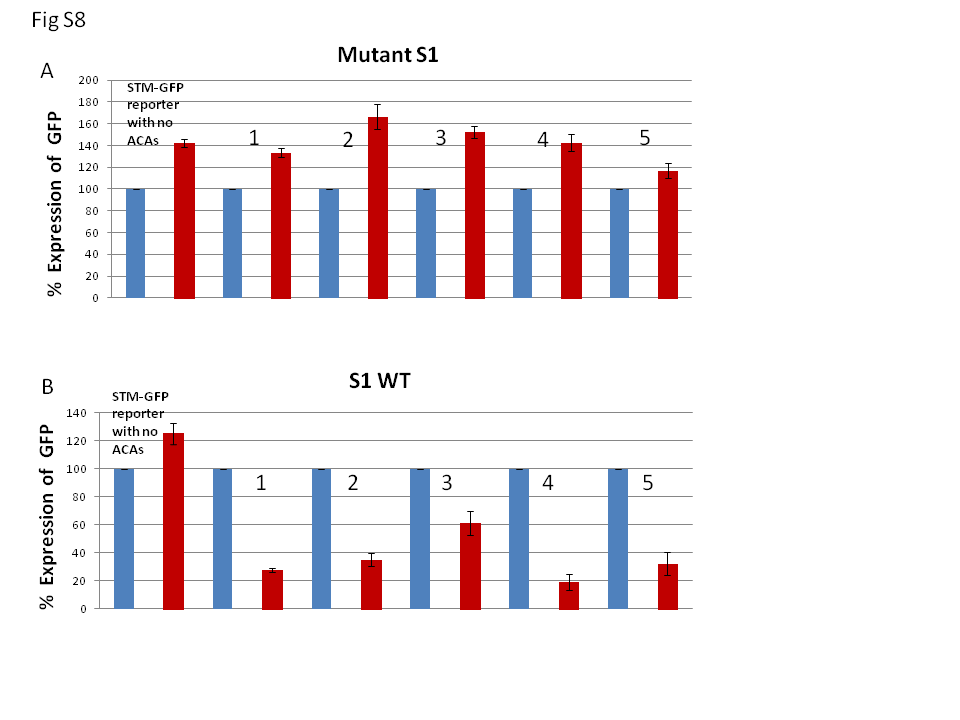

Supplement: Figure S8 — The MazF-induced cleavage of an in-frame ACA site (in all 5 different locations) in the GFP-STM reporter is dependent on the EDF-like sequence in bS1. The experiment was done as described in the legend to Fig. 4. All 5 different GFP-STM in-frame ACA reporters were used. (A) Quantitative comparison of GFP expression in MazF-induced samples (red bars) versus uninduced samples (blue bars) in cells harboring the pUH-C plasmid carrying the gene coding for a mutant bS1. (B) As in panel A with a WT bS1. Download [file mbo006163073sf8.tif]

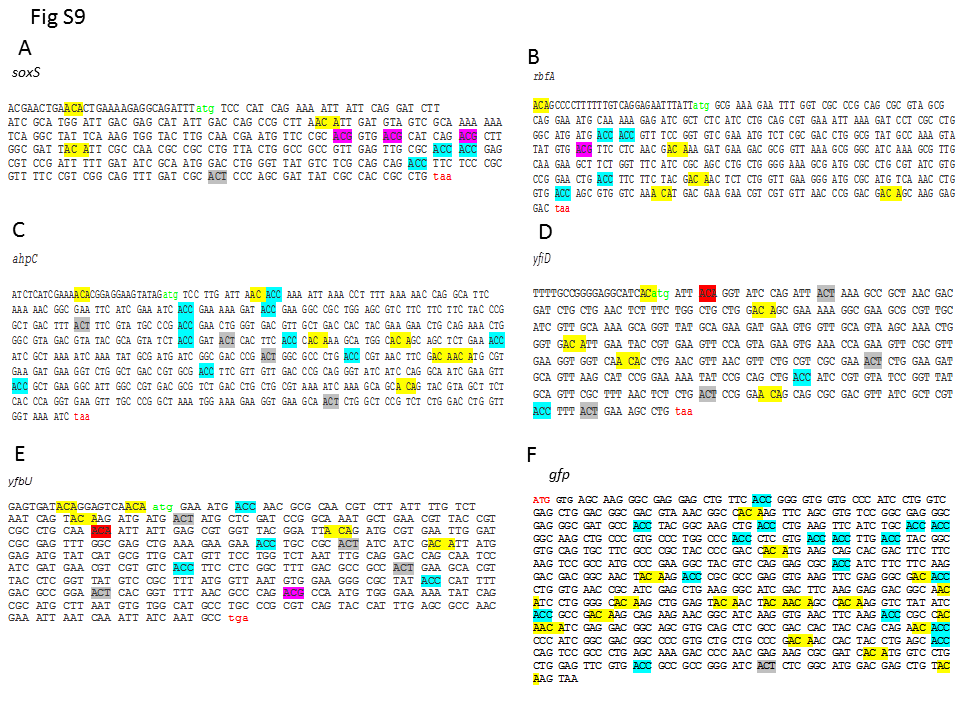

Supplement: Figure S9 — Locations of threonine synonyms ACA, ACC, ACG, and ACT in examples of the genes specifying MazF-induced regulon and in the GFP sequence. DNA sequences are presented: soxS (A), rbfA (B), ahpC (C), yfiD (D), yfbU (E), and gfp (F). Out-of-frame ACA sites are highlighted in yellow, in-frame ACAs are highlighted in red, ACCs are highlighted in blue, ACGs are highlighted in magenta, and ACTs are highlighted in gray. Download [file mbo006163073sf9.tif]
